# Biphasic Calcium Phosphate Versus Demineralized Freeze-Dried Bone Allograft in the Treatment of Periodontal Disease: A Clinical and Radiographical Evaluation

**DOI:** 10.7759/cureus.29131

**Published:** 2022-09-13

**Authors:** Santosh Kumar, Nahid Desai, Surabhi Joshi, Tanvi Hirani, Shreya Gajjar, Chandni Patel, Sushmita R Bhakkand, Gaurav A Girdhar, Sharaschandra R Govindool, Wan Farizatul Shima Wan Ahmad Fakuradzi, Mainul Haque

**Affiliations:** 1 Periodontology and Implantology, Karnavati University, Gandhinagar, IND; 2 Periodontology and Implantology, Karnavati School of Dentistry, Karnavati University, Gandhinagar, IND; 3 Periodontology, Karnavati School of Dentistry, Gandhinagar, IND; 4 Periodontology, Karnavati School of Dentistry, Karnavati University, Gandhinagar, IND; 5 Periodontology, Karnavati University, Gandhinagar, IND; 6 Periodontics and Endodontics, University at Buffalo, New York, USA; 7 Community Medicine, Faculty of Medicine and Defence Health, National Defence University of Malaysia, Kuala Lumpur, MYS; 8 Pharmacology and Therapeutics, National Defence University of Malaysia, Kuala Lumpur, MYS

**Keywords:** alveolar bone, periodontal ligament, progressive destruction, periodontitis, transplant, radiological assessment, demineralized fridge dried bone, bony defect, bone grafts, biphasic calcium phosphate

## Abstract

Aim

The study aimed to clinically and radiographically evaluate the effect of biphasic calcium phosphate (BCP) versus demineralized freeze-dried bone allograft (DFDBA) in treating periodontal disease.

Method

The study consisted of 44 patients. The sites were randomly assigned to receive one of two treatment modalities (BCP at site 1 and demineralized freeze-dried bone at site 2) by a computerized method. All the clinical data were measured with the help of a University of North Carolina-15 (UNC-15) probe at the baseline, three months, and six months postoperatively. Radiovisiographs were taken using a Rinn XCP® (Dentsply/Rinn Corp, Elgin, IL) system and an oral grid using the paralleling technique. A manual calculation of the defect area was undertaken at the end of six months and was compared with the other groups.

Result

The linear bone growth recorded for site 1 at the end of six months was 3.8 ± 1.14 mm, and site 2 was 4.6 ± 1.07 mm. The intergroup comparison showed more remarkable linear bone growth in site 2, which was statistically insignificant, with a mean difference of 0.8 ± 1.23 mm and a *p*-value of 0.07.

Conclusion

Improvements were observed on all the documented parameters. However, the sites treated with DFDBA showed better periodontal regeneration.

## Introduction

Periodontitis is an inflammatory disease of the supporting tissues of the teeth caused by specific micro-organisms or a group of specific micro-organisms, resulting in progressive destruction of the periodontal ligament and alveolar bone with pocket formation recession or both [1]. The pathological hallmark of periodontitis is the destruction of the supporting structures of the involved teeth with loss of attachment.

A decrease in the alveolar bone assistance is the typical symptom of degenerative periodontal diseases, which occurs as the anatomical continuation of the spread of periodontitis in the apical region [2]. Different forms of bone abnormalities result from periodontal diseases [3]. The crucial goal of periodontal management is the preservation of natural dentition in healthy function. Once the attachment apparatus is lost, regenerating the periodontium to its pre-diseased state requires optimal care [4].

Regeneration is the procreation or re-formation of a lost or injured tissue to re-establish the architecture and function of the periodontal tissues [5]. Periodontal regeneration indicates restoring supporting structures like the cementum, alveolus, and periodontal ligament to their original levels (i.e., the levels before periodontal pathogens caused the tissue destruction) [6].

Deep intraosseous defects constitute the most significant challenge for the clinician, often demanding access through flap surgery in alliance with regenerative bone procedures, which incorporate the use of guided tissue regeneration (GTR), bone substitute grafts, and biologic derivatives like enamel matrix proteins (EMPs) and growth factors or a permutation of these techniques. The emergence of various regenerative approaches in periodontics has expanded the patient’s treatment options and augmented the lasting prognosis of multiple teeth with higher periodontal destruction [7].

Contemporary literature suggests that only GTR and osseous grafting have resulted in successful periodontal regeneration [8,9]. The use of bone grafts for reconstructing osseous defects resulting from periodontal disease dates back to 1923 [9], revived by Nabers and O’Leary in 1965 [10]. Bone regeneration in osseous defects requires a structural framework of clot development, maturation, and remodeling, and bone replacement grafts provide this framework [11]. Bone grafts are often used to reduce probing pocket depth, gain clinical attachment level (CAL), and bone fills in osseous defects [12].

Bone grafts can be classified as autogenous, allografts, xenografts, or alloplast. Autogenous bone grafts have been considered the gold standard among all grafts [13]. Inadequate availability of these grafts at the recipient site often leads to a second surgical site, severely limiting their use [5]. Allografts and xenografts are suitable replacements [14,15]. However, demerits such as incomplete resorption have been frequently reported [16].

Allografts such as demineralized freeze-dried bone allografts (DFDBAs) have osteoconductive and osteoinductive properties [17]. They were first used in dentistry and medicine in 1965 [18], but Libin et al. were the first to report DFDBA’s use in human periodontal defects in 1975 [19]. Demineralization with hydrochloric acid (HCl) leads to the release of bone-inductive proteins from the bone matrix; they are known as bone morphogenic proteins (BMPs) (i.e., BMP 7, 4, and 2) and are composed of acidic polypeptides that help to stimulate osteoinduction [20]. They encourage attachment, migration, and osteogenesis of the osteoblastic cell when implanted in well-vascularized bone. They encourage endochondral bone development when embedded in tissues that would otherwise not form bone [21].

As allografts are obtained from different individuals and xenografts from other species, some patients may refuse their use because of personal or religious concerns. To overcome these restraints, alloplastic graft materials, which are inorganic, synthetic, and biocompatible substitutes, can be an alternative to managing intrabony defects (IBDs) [22]. As they are easily accessible, they eliminate donor site morbidity and, unlike allografts and xenografts, pose no risk of disease transmission [7].

Ca_3_(PO_4_)_2_ ceramic is an alloplastic material extensively used for periodontal regeneration. In controlled clinical studies, hydroxyapatite (HA) and β-tricalcium phosphate (β-TCP) have revealed substantial clinical corrections in grafted sites compared to non-grafted sites. Still, HA resorbs slowly, whereas β-TCP resorbs unpredictably faster in biological fluids and may not provide a scaffold for the required duration [23]. Hence, BCP ceramic was developed to control the resorbability of β-TCP by combining it with HA (HA retards the resorbability) and maintaining its osteoconductive property [24]. HA/β-TCP is a new biomaterial composite of medical purity BCP (i.e., 60% HA (100% crystalline) and 40% of TCP’s β form (particulate form)). Preclinically, the results showed that this ratio might allow for better control of bioresorbability, resulting in accelerated new bone formation [25]. However, there are limited controlled clinical trials comparing HA/β-TCP and DFDBA as bone replacement grafts.

Objectives of the study

This study aimed to clinically and radiographically evaluate the effect of BCP versus demineralized freeze-dried bone allograft (DFDBA) in treating intrabony defects. (I) To evaluate the clinical efficacy of BCP alone in intrabony defects. (II) To evaluate the clinical efficacy of DFDBA alone in intrabony defects. (III) To compare the regenerative capacity of both the grafting materials.

## Materials and methods

Study design and patient criteria

This study evaluated and compared the clinical and radiographical parameters between site 1, BCP, and site 2, where DFDBA was used in intrabony defects. This study was a split-mouth, triple-blinded, randomized crossover controlled clinical trial. This study was obtained by the Institutional Review Board (IRB) of Karnavati School of Dentistry, Karnavati University, Gandhinagar, India (Reference No. KSD/KECB/2020/234), and the patients' well-informed about the study design and publication with written informed consent were obtained before any procedure started. Forty-four patients were selected from Karnavati University’s Department of Periodontology and Implantology in Gujarat, India.

Sample Size

**Figure 1:**
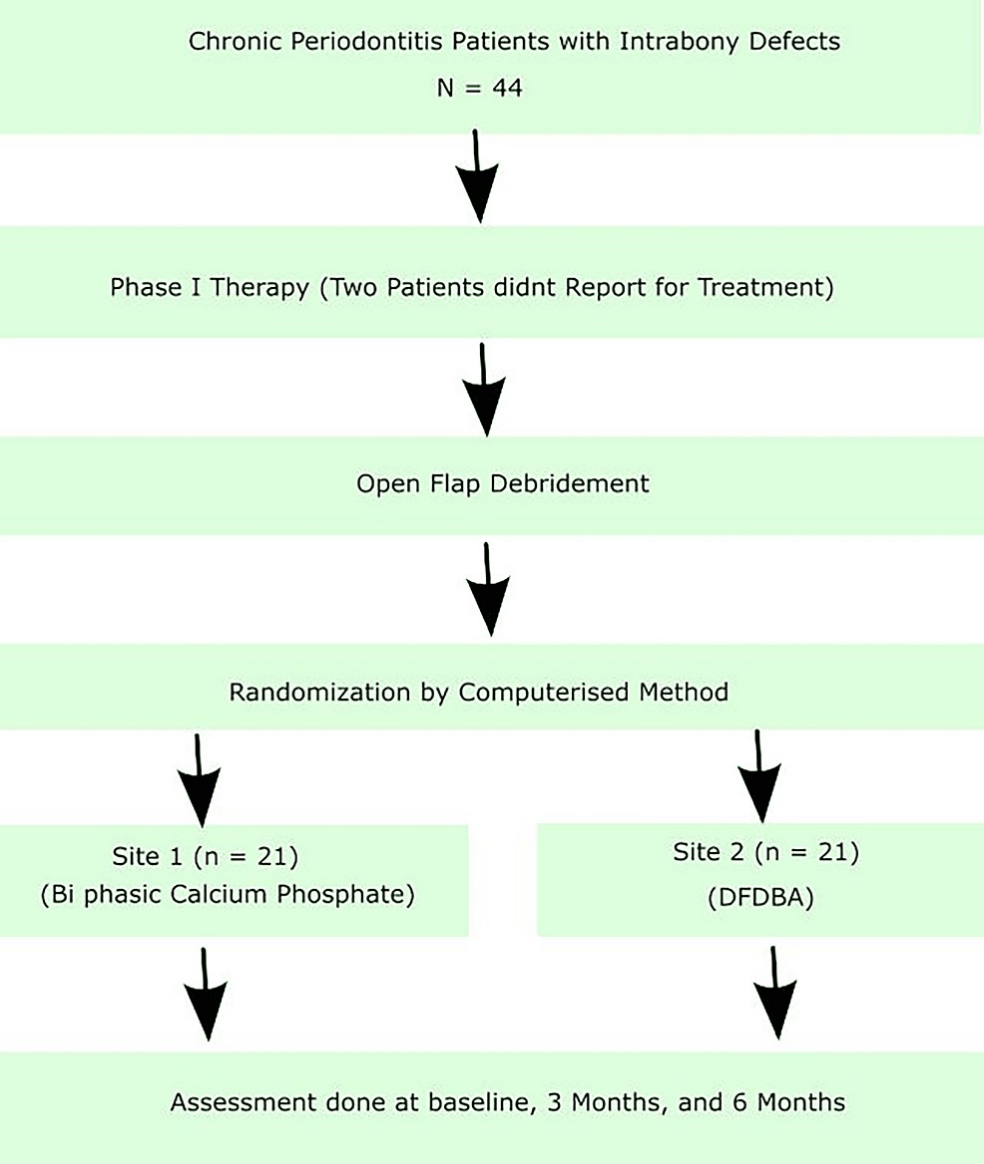
Flow chart. DFDBA: demineralized freeze-dried bone allograft.

The study sample consisted of 44 patients initially, but two patients failed to follow up. Patients who participated in this study were of both sexes. These cases were systemically healthy and diagnosed with generalized chronic periodontitis with deep intrabony bilateral/contralateral defects with a probing depth of ≥5 mm and at least 3 mm as detected on the radiograph. The following formula was used to determine the sample size: n = (Zα/2 + Zβ )2 *2*σ2/d2, where Z α/2 is the critical value of the normal distribution at α/2. Patients who failed to maintain adequate oral hygiene (plaque index >1) after phase. The total calculated sample size was calculated at 40 patients. Still, taking into consideration that few patients will fail to follow up, we included a larger number in our study therapy; pregnant and lactating women, smokers, habitual tobacco consumers, those who did not give consent, and those who failed to follow up were excluded from the study. Additionally, the study sample age range was 30-60 years. The plaque index assesses the amount of dental plaque visible on all teeth' vestibular and lingual surfaces, except the third molars. The sites were randomly assigned to receive either treatment modality by a computerized method (Figure 1).

Site 1: Biphasic Calcium Phosphate

The BCP used here was Meta Bonemedik-DM bone™ (Union Dental, New Delhi, India) in size 0.5-1.0 μm. It contains 60% HA and 40% β-TCP. This fully synthetic bone graft substance is a medically pure BCP composite. This ceramic might be classified as resorbable because of the β-TCP content. The ratio of 60:40 is ideal for new bone formation and osteoconduction. This ratio shows a high uptake of calcium when compared with other combinations of HA/βTCP [24].

Site 2: DFDBA

The DFDBA (TATA, Mumbai, India) particle size 500-1040 μm was used in the present study. It stimulates the immigration, attachment, and osteogenesis of the mesenchymal cells when implanted in highly vascularized bone. It also highly boosts endochondral bone regeneration when inserted into tissues that would otherwise not form bone. DFDBA encompasses bone morphogenic proteins (BMPs) such as BMP 7, 4, and 2, which assist in stimulating osteoinduction. The significant advantage of the DFDBA used here was its affordable cost [26].

The patients who fulfilled the inclusion criteria underwent phase I therapies and were given oral hygiene instructions. At baseline, personal history, dental history, and medical history were recorded. All the clinical records were taken, and an occlusal stent was fabricated for the treatment-targeted tooth. This offered a fixed reference point and angulation for the measurements for any future references at each site during the study period.

Clinical and radiographic parameters

All the scientific parameters like plaque index (PI) (Quigley-Hein plaque index updated by Turesky et al.) [27], modified gingival index (GI) [28], and probing pocket depth (PPD) were recorded. The Turesky modified version of the dental plaque index is as follows: 0 = no plaque, 1 = separate flecks of plaque at the cervical margin of the tooth, 2 = a thin continuous band of plaque (up to 1 mm) at the cervical margin of the tooth, 3 = a band of plaque wider than 1 mm but covering less than one-third of the crown of the tooth, 4 = plaque covering at least one-third but less than two-thirds of the crown of the tooth, 5 = plaque covering two-thirds or more of the crown of the tooth. A University of North Carolina-15 (UNC-15) probe recorded relative attachment level (RAL) with the help of a customized acrylic stent at the baseline, three months, and six months postoperatively.

Radiovisiographs (RVG) were obtained by the Rinn XCP® (Dentsply/Rinn Corp, Elgin, IL) system (paralleling technique) with a standard intraoral grid attached. The total zone of the bony defect was analyzed. The radiographic parameters were documented at the baseline and six months postoperatively. To avoid inter-examiner bias, a single operator (dental surgeon 1) blinded to the study recorded all parameters.

The anatomic landmarks identified by Eickholz et al. [29] were considered for radiographic analysis: (I) cementoenamel junction (CEJ), (II) alveolar crest (AC), and (III) the base of the defect (BD).

**Figure 2:**
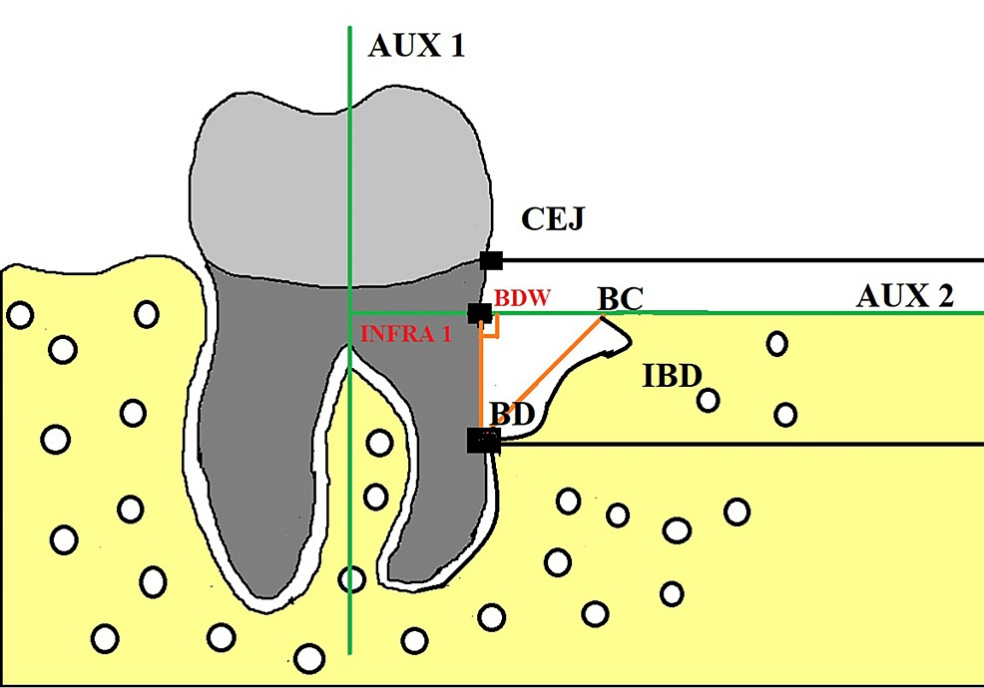
Radiographic landmarks for calculation. Image Credits: Santosh Kumar. BDW: bone defect width, CEJ: cementoenamel junction, BD: base of the defect, BC: bony crest.

BD is the distance from the cementoenamel junction (CEJ) to the most profound extension of the bony defect [30]. The alveolar crest (AC) is the distance from the cementoenamel junction (CEJ) to the alveolar ridge [30]. AUX I is an auxiliary line drawn in the direction of the tooth axis (Figure 2). AUX II is the second auxiliary line perpendicular to the tooth axis, drawn through the most coronal extension of the lateral wall of the intrabony defect. INFRA 1 is the distance from CEJ to BD minus the distance from CEJ to AC (the difference between the distances from CEJ to BD and CEJ to AC). INFRA 2 is the distance from the point where AUX II crosses the contour of the root to BD. Bone defect width (BDW) is the measurement from the defect’s lateral margin to the intersection of the point where AUX II crosses the root’s surface. Linear bone growth is the BD to CEJ at the baseline minus the BD to CEJ after six months. The defect area equals ½ (INFRA 1 × BDW) of the bone fill % (linear bone growth/defect depth) × 100.

Surgical Procedure

The operative area was anesthetized with a 2% lignocaine hydrochloride solution (1:80,000). After achieving adequate anesthesia, infraclavicular incisions were placed using a 15-number blade. Full-thickness mucoperiosteal flaps were elevated on lingual and buccal sides for attaining access to the defect zone. The granulation tissues were detached using Gracey curettes, and accurate scaling and root planning were undertaken. After complete debridement, the osseous defect was measured by a UNC-15 probe. The bone defect width, vertical bone depth, and existing bony walls were recorded.

Pre-suturing and filling with BCP bone grafts occurred on site 1 and DFDBA grafts on site 2. The surgeries were performed on the respective sites at an interval of two weeks. The time difference was calculated from the day the first surgery was performed. Patients were advised not to take any other medicines than prescribed unless an emergency arose. The mucoperiosteal flaps that were pre-sutured were relocated and secured with interrupted sutures. The operating area was shielded with a non-eugenol (Coe-pack). A different single operator (dental surgeon 2) performed all surgeries.

The patients were advised to avoid chewing and brushing in the surgical area for two weeks. They were advised to avoid rinsing, spitting, and consuming hard or hot food for 24 hours. Patients were instructed to use 10 ml of 0.2% chlorhexidine gluconate solution twice daily for mouth rinsing for effective plaque control after the day of surgery.

They were prescribed medications, including 500 mg three times a day (TDS) of amoxicillin for three days and 400 mg TDS of diclofenac sodium for three days. After ten days, patients were recalled for sutures and periodontal dressing removal. The area was carefully inspected for healing. A single physician was allotted for this study, who questioned all the patients for signs and symptoms and also physically examined the patients if required. All the data regarding this was recorded in black and white.

Statistical Analysis

The data gathered were organized and subjected to statistical analysis using SPSS Software (IBM Corp. Released 2013. IBM SPSS Statistics for Windows, Version 22.0. IBM Corp, Armonk, NY). The intragroup comparisons in site 1 and site 2 from baseline to three months and from baseline to six months were made using a student’s paired t-test. Similarly, the intergroup comparison between site 1 and site 2 was made using an independent t-test and analysis of variance (ANOVA). The *p*-value <0.05 was considered statistically significant.

## Results

The study initially consisted of 44 patients, but two patients failed to follow up. The attrition rate was adjusted to 4%. The remaining 42 patients completed the study. The mean age of the study subjects was 43 years. No post-surgical complications or allergic reactions were documented after the surgery, and all the sites displayed flawless healing. Although study respondents had flawless healing, a wound healing index was calculated.

Regarding intragroup comparison, the baseline plaque index score, three-month score, and six-month score were 0.67 ± 0.2, 0.61 ± 0.16, and 0.56 ± 0.2, respectively, with a *p*-value of 0.122, 0.01, and 0.173, respectively. The plaque score was reduced considerably from baseline to six months. The baseline modified gingival index scores, three-month score, and six-month score were 0.88 ± 0.29, 0.75 ± 0.13, and 0.6 ± 0.16 with p-values of 0.109, 0.008, and 0.015, respectively (Table 1). The gingival score dwindled considerably from baseline to six months and from three months to six months.

**Table 1:** Intragroup comparison of plaque index and modified gingival index at baseline and six months. *Statistically significant. Mean of plaque of index (PI)/modified gingival index (MGI) ± standard deviation (SD).

Groups	Time	N	Mean ± SD	P-value
PI	Baseline	21	0.67 ± 0.20	0.122
	Three months	21	0.61 ± 0.16	
	Baseline	21	0.67 ± 0.20	0.01*
	Six months	21	0.56 ± 0.20	
MGI	Baseline	21	0.88 ± 0.29	0.109
	Three months	21	0.75 ± 0.13	
	Baseline	21	0.88 ± 0.29	0.008*
	Six months	21	0.60 ± 0.16	

The pocket probing depth (in mm) of the intragroup comparison for site 1 was 8.4 ± 1.35 at baseline. At six months, it reduced to 4.8 ± 0.79, showing a substantial reduction in probing depth with a mean difference of 3.6 ± 1.07 and a p-value of <0.001, which is statistically significant. For site 2, the PPD (in mm) documented at baseline and six months was 8.8 ± 1.32 and 3.7 ± 0.48, respectively, showing a mean difference of 5.1 ± 1.29 from baseline to six months and a *p*-value of <0.001, which is statistically significant (Table 2).

**Table 2:** Intragroup comparison of pocket probing depth values (in mm) of sites 1 and 2 at baseline and six months. *Statistically significant. Mean of site 1/2 ± standard deviation (SD).

Site	Time	N	Mean ± SD	Mean difference ± SD	P-value
Site 1 (biphasic Ca_3_(PO_4_)_2_)	Baseline	21	8.4 ± 1.35	2.5 ± 0.97	<0.001*
	Three months	21	5.9 ± 0.88		
	Baseline	21	8.4 ± 1.35	3.6 ± 1.07	<0.001*
	Six months	21	4.8 ± 0.79		
Site 2 (DFDBA)	Baseline	21	8.8 ± 1.32	3.6 ± 1.58	<0.001*
	Three months	21	5.2 ± 0.79		
	Baseline	21	8.8 ± 1.32	5.1 ± 1.29	<0.001*
	Six months	21	3.7 ± 0.48		

Regarding the intergroup comparison between sites 1 and 2 after the t-test, the PD was statistically non-significant at baseline and three months, given that the p-values were 0.479 and 0.089, respectively. However, at six months, the PD reduction was statistically significant in site 2 than at site 1, with a *p*-value of 0.003 (Table 3).

**Table 3:** Intergroup comparison of pocket probing depth values (in mm) of sites 1 and 2, baseline, and six months. *Statistically significant; ns: non-significant. Mean ± standard deviation (SD).

Time	Site	N	Mean ± SD	Mean differences ± SD	p
Baseline	Site 1 (biphasic Ca_3_(PO_4_)_2_)	21	8.4 ± 1.35	−0.4 ± 1.71	0.479^ns^
	Site 2 (DFDBA)	21	8.8 ± 1.32		
Three months	Site 1 (biphasic Ca_3_(PO_4_)_2_)	21	5.9 ± 0.88	0.7±1.16	0.089^ns^
	Site 2 (DFDBA)	21	5.2 ± 0.79		
Six months	Site 1 (biphasic Ca_3_(PO_4_)_2_)	21	4.8 ± 0.79	1.1 ± 0.88	0.003*
	Site 2 (DFDBA)	21	3.7 ± 0.48		

The RAL (in mm) on intragroup comparison for Site 1 at baseline was 10 ± 1.15, which decreased to 8.4 ± 1.07 at 3 months and 7.4 ± 1.17 at 6 months. For Site 2, the RAL (in mm) at baseline, 3 months, and 6 months were 10.1 ± 1.37, 7.1 ± 0.99, and 5.4 ± 0.84, respectively, which were significant with p-values of <0.001. 

On intergroup comparison between Site 1 and Site 2 after the t-test, the relative attachment level was statistically non-significant at baseline, as the p-value was 0.853. However, at 3 months and 6 months, the RAL reduction was statistically significant in Site 2 than in Site 1, with *p*-values of 0.009 and <0.001, respectively (Table 4).

**Table 4:** Intergroup comparison of relative attachment level values (in mm) of sites 1 and 2 at baseline, three, and six months. *Statistically significant; ns: non-significant. Mean ± standard deviation (SD). DFDBA: demineralized freeze-dried bone allograft.

Time	Site	N	Mean ± SD	Mean difference ± SD	p
Baseline	Site 1 (biphasic calcium phosphate)	21	10 ± 1.15	−0.1 ± 1.66	0.853^ns^
	Site 2(DFDBA)	21	10.1 ± 1.37		
Three months	Site 1 (biphasic calcium phosphate)	21	8.4 ± 1.07	1.3 ± 1.25	0.009*
	Site 2(DFDBA)	21	7.1 ± 0.99		
Six months	Site 1 (biphasic calcium phosphate)	21	7.4 ± 1.17	2.0 ± 0.82	<0.001*
	Site 2(DFDBA)	21	5.4 ± 0.84		

The linear bone growth recorded for site 1 at the end of six months was 3.8 ± 1.14 and 4.6 ± 1.07 for site 2. The intergroup comparison showed more remarkable linear bone growth in site 2, which was statistically non-significant with a mean difference of 0.8 ± 1.23 and a *p*-value of 0.07.

**Figure 3:**
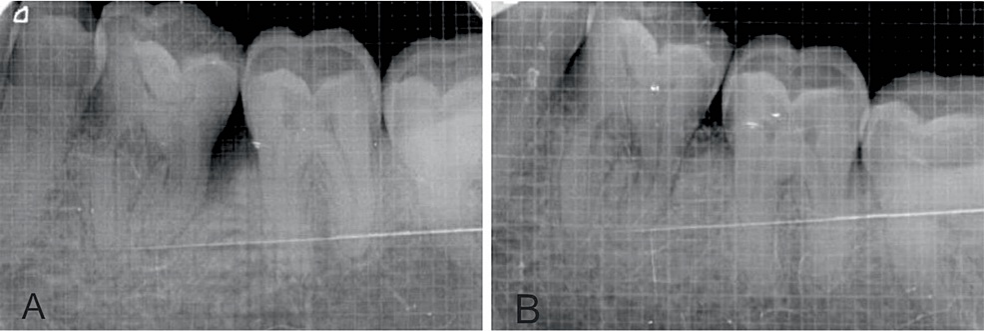
(A) Radiograph showing the bone level at baseline at site one (BCP). (B) Radiograph showed the bone level at six months at site one (BCP). BCP: biphasic calcium phosphate.

**Figure 4:**
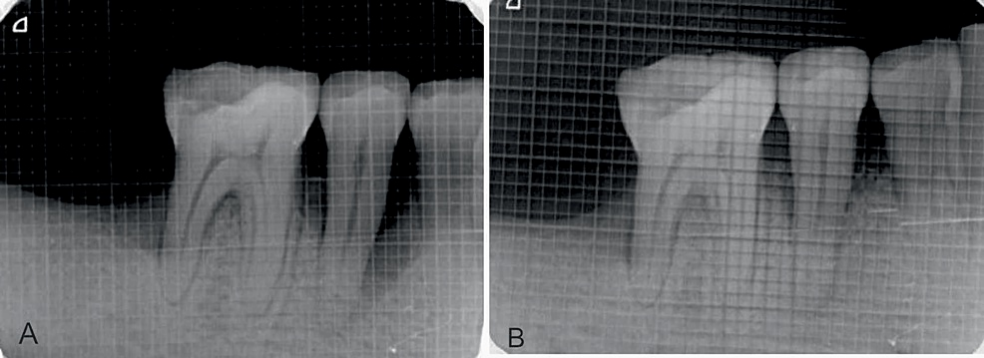
(A) Radiograph shows the baseline bone level at site two (DFDBA). (B) Radiograph showed the bone level at six months at site two (DFDBA). DFDBA: demineralized freeze-dried bone allografts.

The percentage of bone fill recorded for site 1 at the end of six months (Figures 3A, 3B) was 56.29 ± 11.17 and 63.38 ± 8.78 for site 2 (Figures 4A, 4B). The intergroup comparison showed a statistically significant greater percentage of bone fill in site 2, with a mean difference of 7.09 ± 8.37 and a p-value of 0.025 (Table 5).

**Table 5:** Intergroup comparison of the percentage of bone fill at six months. *Statistically significant.

	N	Mean ± SD	Mean difference ± SD	p
Site 1 (biphasic Ca_3_(PO_4_)_2_)	21	56.29 ± 11.17	−7.09 ± 8.37	0.025*
Site 2 (DFDBA)	21	63.38 ± 8.78		

## Discussion

Deep periodontal pockets coupled with osseous lesions characterize the specific risk factors for or indicators of disease progression [31]. Thus, treating them to halt the progression of periodontal disease is of prime importance to the tooth. Bony lesions often require access to flap surgery unaided or in combination with bone resective or regenerative techniques; they may not be available for periodontal debridement through non-surgical therapy [32].

After periodontal flap surgery, the unpredictability of osseous defect fill has investigated various bone grafting materials. The autogenous bone graft is considered the gold standard. Still, the limitation of the number of bone grafts available and the morbidity associated with the second surgical site for graft procurement are drawbacks [12]. This material is recognized in the 1996 World Workshop in Periodontics consensus report to fulfill all criteria for the promotion of periodontal regeneration [33]. The disadvantages of DFDBA are cost, lack of patient acceptance due to fear of disease transfer from the donor, and radiolucency [34].

Alloplasty overcomes some of the disadvantages of autogenous and allogenic bone grafts [35]. BCP has osteoconductive properties and may be appropriate as a bone graft material [13]. The capability of BCP to be bio-absorbed and to form a direct tough bond with the host bone also makes it a better choice as a bone graft. In the histological sections, it was observed that it completely integrated into a secondarily formed spongy bone. This resulted in complete osseous regeneration of the former defect area [36].

The present study was a split-mouth, triple-blinded, randomized crossover controlled clinical trial. The randomized controlled trial (RCT) is often considered the gold standard for a clinical trial. It offers the advantage of minimizing allocation bias and matching both known and unknown prognostic factors in the assignment of treatments. A split-mouth design was applied in this study to minimize the bias theoretically determined by differences in individual elements, avoid natural variation between individuals, and limit patient-based and defect-based factors. In our research, plaque and gingival index showed improvement from baseline to six months. This could be attributed to an improvement in the home care and reinforcement of oral hygiene instructions at each recall visit.

Our study’s PI at baseline, three months, and six months was 0.67 ± 0.2, 0.61 ± 0.16, and 0.56 ± 0.2, respectively. The plaque score was reduced from baseline to three months and three months to six months, but it was not statistically significant. The plaque score decreased significantly from baseline to six months. The insignificant quantity of plaque did not hamper the regeneration, and all patients maintained appropriate oral hygiene during the study stage. The deterioration in PI scores throughout the study could be attributed to an improvement in home care and an emphasis on oral hygiene instructions at each recall visit. A comparable tendency of a declining plaque index was observed in a study by Franco and Rubia [37] and Agarwal et al. [38]. The modified gingival index scores at baseline in the study were 0.88 ± 0.29, which reduced to 0.75 ± 0.13 at three months and, significantly, to 0.6 ± 0.16 at six months. This is in agreement with an investigation by Stein et al. [39].

The pocket probing depth at sites treated with BCP showed a significant decrease at the end of six months. Previous research observed similar results [7,39-41]. Similarly, the pocket probing depth at sites treated with DFDBA also showed a significant decrease at the end of six months. The current study results align with earlier studies [42-46]. But the sites treated with DFDBA showed a better improvement when compared to the sites treated with BCP.

One of the most important parameters for assessing periodontal destruction is the loss of connective tissue attachment to the tooth root surface. The “gold standard” for recording changes in periodontal status is a longitudinal measurement of CAL from the CEJ or a RAL from a fixed reference point. The assessment of attachment levels using fixed reference points provides better information relating to gain or loss of attachment to the root surface and in assessing disease progression compared with pocket depth measurement.

The RAL at the sites treated with BCP showed a significant gain at six months. The present study results follow the findings of earlier studies [7,39-41,47]. The RAL at the sites treated with DFDBA also showed a significant gain at six months. These results are similar to previous studies [45,46,48-50]. The DFDBA grafted sites showed better gains in RAL than the BCP grafted sites.

The linear bone growth recorded for site 1 at the end of six months was 3.8 ± 1.14 and 4.6 ± 1.07 for site 2. The intergroup comparison of site 2 showed comparatively higher linear bone growth, which is statistically non-significant. The results are analogous to those in the studies of Aspriello et al. [51] and Jayakumar et al. [52], which examined enamel matrix derivative (EMD) and demineralized freeze-dried bone allografts (DFDBA) with DFDBA alone for the treatment of defects. The results are also similar to an animal study conducted by El-Dien et al. [53], in which bilateral defects were arbitrarily treated with DFDBA + platelet-rich plasma (PRP) (test) or DFDBA alone (control). The bone fill at site 2 (DFDBA) was 4.6 ± 1.07 mm at six months, which is in line with previous studies [45,46,48-50,54].

The percentage of bone fill recorded for site 1 at the end of six months was 56.29 ± 11.17 and 63.38 ± 8.78 for site 2. The intergroup comparison showed a more significant percentage of bone fill in site 2, which was statistically significant. Similar results were observed in a study done by El-Dien et al. [53].

DFDBA contains BMPs that help stimulate osteoinduction by: (1) acting as mitogens on undifferentiated mesenchymal cells and osteoblast precursors; (2) inducing the expression of the osteoblast phenotype (e.g., increasing alkaline phosphatase activity in bone cells); and (3) acting as chemo-attractants for mesenchymal cells and monocytes as well as binding to extracellular matrix type IV collagen [55]. This may be the reason behind the more significant percentage of bone fill in site 2 in the present study.

The current study was a cross-sectional study with its inherent limitations. Thereby incapable of determining the incidence, studying uncommon health disorders, and making causal inferences. This single-center study was conducted at the Department of Periodontology, School of Dentistry, Karnavati University, India. Thereafter, it is difficult to generalize the findings all over the country.

## Conclusions

With the extensive array of grafting biomaterials currently available, clinicians often find themselves lost in selecting the best option. This study is intended to make the selection procedure easier for dental clinicians. This study's findings had the potential to conclude the following: (I) The plaque and gingival index decreased in both groups, which indicates an overall improvement in the oral hygiene status of the patients after treatment. (II) Healing was uneventful, with absolutely no allergic reactions, which shows that both the graft materials were safe for human use. (III) Reduction in pocket depth was more in the sites treated by DFDBA, which offers a significant regenerative potential of DFDBA. (IV) Radiographic parameters, i.e., area of defects, linear bone growth, and percentage of bone fill, were more in the sites treated with DFDBA. This signifies the better regenerative potential of DFDBA. Although this study has certain limitations, it can be concluded that the use of DFDBA for treating intrabony defects is more beneficial when compared to BCP.

## References

[1] Saini R, Marawar PP, Shete S, Saini S (2009). Periodontitis, a true infection. J Glob Infect Dis.

[2] Könönen E, Gursoy M, Gursoy UK (2019). Periodontitis: a multifaceted disease of tooth-supporting tissues. J Clin Med.

[3] Amaranath BJ, Das N, Gupta I, Gupta R, John B, Devi MP (2020). Types of bone destruction and its severity in chronic periodontitis patients with tobacco smoking habit using periapical radiographs and transgingival probing: a cross-sectional study. J Indian Soc Periodontol.

[4] Zander HA, Polson AM, Heijl LC (1976). Goals of periodontal therapy. J Periodontol.

[5] Caton JG (1997). Overview of clinical trials on periodontal regeneration. Ann Periodontol.

[6] Liu J, Ruan J, Weir MD (2019). Periodontal bone-ligament-cementum regeneration via scaffolds and stem cells. Cells.

[7] Lee MJ, Kim BO, Yu SJ (2012). Clinical evaluation of a biphasic calcium phosphate grafting material in the treatment of human periodontal intrabony defects. J Periodontal Implant Sci.

[8] Nyman S, Lindhe J, Karring T, Rylander H (1982). New attachment following surgical treatment of human periodontal disease. J Clin Periodontol.

[9] Sumer M, Keles GC, Cetinkaya BO, Balli U, Pamuk F, Uckan S (2013). Autogenous cortical bone and bioactive glass grafting for treatment of intraosseous periodontal defects. Eur J Dent.

[10] Nabers CL, O'Leary TJ (1965). Autogenous bone transplants in the treatment of osseous defects. J Periodontol.

[11] Hazari V, Choudhary A, Mishra R, Chandrashekar KT, Trivedi A, Pathak PK (2021). Clinical and radiographic analysis of novabone putty with platelet-rich fibrin in the treatment of periodontal intrabony defects: a randomized control trial. Contemp Clin Dent.

[12] Zhao R, Yang R, Cooper PR, Khurshid Z, Shavandi A, Ratnayake J (2021). Bone grafts and substitutes in dentistry: a review of current trends and developments. Molecules.

[13] Titsinides S, Agrogiannis G, Karatzas T (2019). Bone grafting materials in dentoalveolar reconstruction: a comprehensive review. Jpn Dent Sci Rev.

[14] Camelo M, Nevins ML, Schenk RK, Simion M, Rasperini G, Lynch SE, Nevins M (1998). Clinical, radiographic, and histologic evaluation of human periodontal defects treated with Bio-Oss and Bio-Gide. Int J Periodontics Restorative Dent.

[15] Bowers GM, Chadroff B, Carnevale R (1989). Histologic evaluation of new attachment apparatus formation in humans. Part II. J Periodontol.

[16] Caplanis N, Lee MB, Zimmerman GJ, Selvig KA, Wikesjö UM (1998). Effect of allogeneic freeze-dried demineralized bone matrix on regeneration of alveolar bone and periodontal attachment in dogs. J Clin Periodontol.

[17] Sethi AK, Kar IB, Mohanty T, Mishra N, Singh AK (2018). Use of plasma-enriched demineralized freeze-dried bone matrix in postsurgical jaw defects. Natl J Maxillofac Surg.

[18] Jaiswal Y, Kumar S, Mishra V, Bansal P, Anand KR, Singh S (2017). Efficacy of decalcified freeze-dried bone allograft in the regeneration of small osseous defect: a comparative study. Natl J Maxillofac Surg.

[19] Libin BM, Ward HL, Fishman L (1975). Decalcified, lyophilized bone allografts for use in human periodontal defects. J Periodontol.

[20] Shigeyama Y, D'Errico JA, Stone R, Somerman MJ (1995). Commercially-prepared allograft material has biological activity in vitro. J Periodontol.

[21] Wu J, Liu Y, Cao Q, Yu T, Zhang J, Liu Q, Yang Xl (2020). Growth factors enhanced angiogenesis and osteogenesis on polydopamine-coated titanium surface for bone regeneration. Mater Des.

[22] Fernandez de Grado G, Keller L, Idoux-Gillet Y (2018). Bone substitutes: a review of their characteristics, clinical use, and perspectives for large bone defects management. J Tissue Eng.

[23] Nery EB, Lee KK, Czajkowski S (1990). A veterans administration cooperative study of biphasic calcium phosphate ceramic in periodontal osseous defects. J Periodontol.

[24] Nery EB, LeGeros RZ, Lynch KL, Lee K (1992). Tissue response to biphasic calcium phosphate ceramic with different ratios of HA/beta TCP in periodontal osseous defects. J Periodontol.

[25] Sculean A, Windisch P, Szendröi-Kiss D (2008). Clinical and histologic evaluation of an enamel matrix derivative combined with a biphasic calcium phosphate for the treatment of human intrabony periodontal defects. J Periodontol.

[26] Gajiwala AL, Kumar BD, Chokhani P (2007). Evaluation of demineralised, freeze-dried, irradiated bone allografts in the treatment of osseous defects in the oral cavity. Cell Tissue Bank.

[27] Turesky S, Gilmore ND, Glickman I (1970). Reduced plaque formation by the chloromethyl analog of victamine C. J Periodontol.

[28] Lobene RR, Weatherford T, Ross NM, Lamm RA, Menaker L (1986). A modified gingival index for use in clinical trials. Clin Prev Dent.

[29] Eickholz P, Hörr T, Klein F, Hassfeld S, Kim TS (2004). Radiographic parameters for prognosis of periodontal healing of infrabony defects: two different definitions of defect depth. J Periodontol.

[30] Eickholz P, Riess T, Lenhard M, Hassfeld S, Staehle HJ (1999). Digital radiography of interproximal bone loss; validity of different filters. J Clin Periodontol.

[31] Monsarrat P, Bernard D, Marty M (2022). Systemic periodontal risk score using an innovative machine learning strategy: an observational study. J Pers Med.

[32] Bertoldi C, Generali L, Cortellini P (2021). Influence of tooth-brushing on early healing after access flap surgery: a randomized controlled preliminary study. Materials (Basel).

[33] Kao RT, Curtis DA, Kim DM (2020). American Academy of Periodontology best evidence consensus statement on modifying periodontal phenotype in preparation for orthodontic and restorative treatment. J Periodontol.

[34] Donovan TE, Marzola R, Murphy KR (2018). Annual review of selected scientific literature: a report of the Committee on Scientific Investigation of the American Academy of Restorative Dentistry. J Prosthet Dent.

[35] Um IW, Ku JK, Kim YM, Yun PY, Chang NH, Kim YK, Choi Y (2020). Allogeneic demineralized dentin matrix graft for guided bone regeneration in dental implants. Appl Sci.

[36] Zhang Y, Wu D, Zhao X (2020). Stem cell-friendly scaffold biomaterials: applications for bone tissue engineering and regenerative medicine. Front Bioeng Biotechnol.

[37] Rodríguez Franco NI, Moral de la Rubia J (2020). Plaque index, oral hygiene habits, and depressive symptomatology as predictors of clinical attachment loss: a pilot study. Int J Dent.

[38] Agarwal A, Manjunath RG, Sethi P, Shankar GS (2019). Platelet-rich fibrin in combination with decalcified freeze-dried bone allograft for the management of mandibular degree II furcation defect: a randomised controlled clinical trial. Singapore Dent J.

[39] Stein JM, Fickl S, Yekta SS, Hoischen U, Ocklenburg C, Smeets R (2009). Clinical evaluation of a biphasic calcium composite grafting material in the treatment of human periodontal intrabony defects: a 12-month randomized controlled clinical trial. J Periodontol.

[40] Pandit N, Gupta R, Gupta S (2010). A comparative evaluation of biphasic calcium phosphate material and bioglass in the treatment of periodontal osseous defects: a clinical and radiological study. J Contemp Dent Pract.

[41] Bansal R, Patil S, Chaubey KK, Thakur RK, Goyel P (2014). Clinical evaluation of hydroxyapatite and β-tricalcium phosphate composite graft in the treatment of intrabony periodontal defect: a clinico-radiographic study. J Indian Soc Periodontol.

[42] Saini AK, Tewari S, Narula SC, Sharma RK, Tanwar N, Sangwan A (2020). Comparative clinical and radiographic evaluation of demineralized freeze-dried bone allograft with and without decortication in the treatment of periodontal intrabony defects: a randomized controlled clinical study. Quintessence Int.

[43] Vaid T, Kumar S, Mehta R, Shah S, Joshi S, Bhakkand S, Hirani T (2021). Clinical and radiographic evaluation of demineralized freeze-dried bone allograft with concentrated growth factor versus concentrated growth factor alone in the treatment of intrabony defects. Med Pharm Rep.

[44] Liu Y, Sun X, Yu J (2019). Platelet-rich fibrin as a bone graft material in oral and maxillofacial bone regeneration: classification and summary for better application. Biomed Res Int.

[45] Gothi R, Bansal M, Kaushik M, Khattak BP, Sood N, Taneja V (2015). A comparative evaluation of freeze dried bone allograft and decalcified freeze dried bone allograft in the treatment of intrabony defects: a clinical and radiographic study. J Indian Soc Periodontol.

[46] Shah M, Patel J, Dave D, Shah S (2015). Comparative evaluation of platelet-rich fibrin with demineralized freeze-dried bone allograft in periodontal infrabony defects: a randomized controlled clinical study. J Indian Soc Periodontol.

[47] Kaushal S, Kapoor A, Singh P, Kochhar G, Khuller N, Basavaraj P (2014). Evaluation of OSSIFI® as alloplastic bone graft material in treatment of periodontal infrabony defects. J Clin Diagn Res.

[48] Rummelhart JM, Mellonig JT, Gray JL, Towle HJ (1989). A comparison of freeze-dried bone allograft and demineralized freeze-dried bone allograft in human periodontal osseous defects. J Periodontol.

[49] Hoidal MJ, Grimard BA, Mills MP, Schoolfield JD, Mellonig JT, Mealey BL (2008). Clinical evaluation of demineralized freeze-dried bone allograft with and without enamel matrix derivative for the treatment of periodontal osseous defects in humans. J Periodontol.

[50] Katuri KK, Kumar PJ, Swarna C, Swamy DN, Arun KV (2013). Evaluation of bioactive glass and demineralized freeze dried bone allograft in the treatment of periodontal intraosseous defects: a comparative clinico-radiographic study. J Indian Soc Periodontol.

[51] Aspriello SD, Ferrante L, Rubini C, Piemontese M (2011). Comparative study of DFDBA in combination with enamel matrix derivative versus DFDBA alone for treatment of periodontal intrabony defects at 12 months post-surgery. Clin Oral Investig.

[52] Jayakumar A, Rajababu P, Rohini S (2011). Multi-centre, randomized clinical trial on the efficacy and safety of recombinant human platelet-derived growth factor with β-tricalcium phosphate in human intra-osseous periodontal defects. J Clin Periodontol.

[53] El-Dien AMS, Fathy S, El-din YA (2021). Potential bone regenerative effects of DFDBA, simvastatin and platelet rich fibrin, radiographically and histologically of intra-bony periodontal defects in white New Zealand rabbits. Open Access Maced J Med Sci.

[54] Budhiraja S, Bhavsar N, Kumar S, Desai K, Duseja S (2012). Evaluation of calcium sulphate barrier to collagen membrane in intrabony defects. J Periodontal Implant Sci.

[55] Winkler J, Abisoye-Ogunniyan A, Metcalf KJ, Werb Z (2020). Concepts of extracellular matrix remodelling in tumour progression and metastasis. Nat Commun.

